# Factors influencing the utilisation of National health insurance program in urban areas of Nepal: Insights from qualitative study

**DOI:** 10.1371/journal.pgph.0003538

**Published:** 2024-07-26

**Authors:** Sushmita Ghimire, Sailaja Ghimire, Devendra Raj Singh, Reshu Agrawal Sagtani, Sudarshan Paudel

**Affiliations:** 1 Department of Public Health, Asian College for Advance Studies, Purbanchal University, Lalitpur, Nepal; 2 School of Public Health, Patan Academy of Health Sciences, Lalitpur, Nepal; 3 Nepal Health Research Council, Kathmandu, Nepal; 4 School of Human and Health Sciences, University of Huddersfield, Huddersfield, United Kingdom; Indian Council of Medical Research - National Institute of Epidemiology, INDIA

## Abstract

Health insurance has been recognised as a crucial policy measure to enhance citizens’ well–being by reducing the financial burden globally. Nepal has also adopted this scheme to support achieving universal health coverage. Various factors influence the overall performance of the program in Nepal. However, there is a lack of evidence on how different factors have influenced the insurance program in the Nepalese context. Therefore, this study aims to explore facilitators and barriers to the utilisation of national health insurance services among service users and other stakeholders. A qualitative study was conducted by interviewing both demand-side participants and supply-side participants in the Bhaktapur District of Nepal. Thematic network analysis was used to analyse data using RQDA software. The socio-ecological model guides the presentation of the identified factors. The study followed the COREQ guidelines to ensure standard reporting of the results. Factors that encourage the use of health insurance services involve individual, community, and policy-related factors. These factors encompass changes in seeking treatment, assistance during enrollment and renewal by enrollment assistant, proximity to the initial point of contact for care, and policy features like individual cards, contribution amount and cashless treatment system. Likewise, lack of physical infrastructure, poor staff management, long waiting times, poor medicine availability, and delays in budget reimbursement were perceived as organisational barriers. At the interpersonal level, obstacles encompass challenges related to staff behaviour, interpersonal relationships, and the information provided by service providers. Identified health services delivery barriers at different levels emphasised the critical need for improving the quality of healthcare and services delivery mechanisms. Overcoming these obstacles is essential for realising health insurance scheme objectives and progressing toward Universal Health Coverage (UHC).

## Introduction

Health insurance programs have a pivotal role in ensuring fair access to high-quality health services worldwide. These programs serve as a means for individuals to overcome financial barriers and secure essential medical care [1]. They are instrumental in achieving Universal Health Coverage by distributing the financial risks associated with healthcare expenses and enhancing access to essential health services [2,3]. Within intricate healthcare systems, health insurance financing is a vital foundation, encompassing three key functions: revenue collection, risk pooling, and service procurement. Risk pooling, a crucial element, enhances health outcomes, boosts productivity, and alleviates financial burdens [4]. Globally, four intertwined risk pooling mechanisms exist in health insurance systems, shaped by factors like income, health requirements, and administrative capacities. These include the state-funded approach, social health insurance, community-based health insurance, and voluntary health insurance systems [3].

Globally, 44 million households suffer financial catastrophes due to out-of-pocket expenditure in health care, resulting in a yearly increase of 25 million households being pushed into a state of extreme poverty [5,6]. The Global Monitoring Report on Financial Protection 2021 showed that around 1.4 billion individuals were facing severe or impoverishing expenses on health care. Additionally, the same report indicated that in Nepal, the rate of catastrophic healthcare expenditure was 10%, and 25% of household consumption or income was recorded at 10.7% and 2.1%, respectively [7]. Out-of-pocket expenditure has a larger role in health financing in various low and middle-income countries as their health system has always been underfunded. As a consequence, households face difficulty in accessing healthcare services or expose themselves to the risk of poverty because of catastrophic expenditures for healthcare [3,8,9]. The significance of health insurance programs becomes even more pronounced in Low- and Middle-Income Countries (LMICs) [10]. Such nations often struggle with inadequate public funding for healthcare services, leaving large proportions of their populations vulnerable to financial stress when seeking medical treatment [11]. Health insurance programs hold the potential to bridge this gap, offering financial safeguards and enriching healthcare access for individuals who would otherwise face significant obstacles. The establishment of robust health insurance systems in LMICs is believed to advance the broader global objective of achieving Universal Health Coverage (UHC) and embracing the principles of Primary Health Care (PHC) [12–14]. However, resource-poor countries like Nepal face significant challenges in providing comprehensive healthcare to their citizens [15,16].

### Nepal’s National health insurance scheme

The adoption of health insurance programs in Nepal offers promise in addressing these challenges by providing a systematic approach to healthcare financing and service provision [17,18]. As Nepal undergoes a transformation towards a federal democratic republic, its healthcare system is evolving, creating an opportune time to explore the dynamics of health insurance utilisation [16,19]. Nepal’s Social Health Insurance Program employs a hybrid approach, integrating elements from diverse mechanisms to attain universal coverage. This approach underscores community, household, and government involvement in its pursuit of equitable health financing within an LMIC context [18,20].

The Nepalese government introduced the Social Health Security Scheme in 2015 to shield people from catastrophic health expenses. This family and contribution-based program offers subsidies for specific groups, and services are accessible through both public and private health facilities via a cashless mechanism [18,21]. A total of 440 service locations, including primary health centres, government hospitals, private hospitals, and community hospitals, are registered as service providers with the Health Insurance Board (an agency for purchasing health care services) to provide these services. These health services are provided across all three tiers of the healthcare system, encompassing local levels, primary healthcare centres, and district and provincial hospitals for secondary care, and specialised services are rendered through the tertiary level of the healthcare system [22].

Health insurance schemes in Nepal typically include a range of services such as outpatient care (including doctor’s visits, diagnostics, and treatments), inpatient coverage (hospital staying costs, surgeries), emergency medical care, medication coverage, maternity and childbirth services, preventive care, laboratory and diagnostic tests, specialist consultations, dental and vision care and rehabilitation services (e.g., some physical and occupational therapy) those are listed in benefit packages [23,24]. Despite enrolling 7.1 million people by August 2023, [25] low enrollment and utilisation rates persist due to challenges in accessing healthcare facilities and dissatisfaction with service providers [17,26].

Numerous previous studies have indicated the low enrollment and utilisation rates of the National Health Insurance Program [27,28]. Literature shows mixed results regarding obstacles and enablers to the utilisation of health insurance programs, particularly in districts outside the capital city and relatively poor accessibility of health services regions [7,29]. Showing the mixed perception among the insures, factors such as limited drug availability, inadequate coverage of the services, long waiting lines, contradictory Act sections, a lack of Health Insurance Board’s organisational standards, inadequately specified National Health Insurance implementing guidelines, and inadequate human resources, etc. are identified barriers leading to the dissatisfaction and reduced financial burden, expanded coverage and better access to the health care services as enabling factors for utilisation [27,30]. However, a research gap exists in providing insight into a holistic understanding of the health insurance program utilisation dynamics in Nepal. The majority of research carried out in Nepal has been limited to investigating the variables affecting health insurance program enrollment and dropout and less explored the factors influencing the program in at wider context [31,32]. In light of these considerations, this study aimed to explore the factors that facilitate or hinder the utilisation of the health insurance program in Nepal. By understanding the dynamics that encourage or impede its use, this study can contribute to the refinement of Nepal’s healthcare system, support its journey towards UHC, and strengthen its commitment to providing accessible and comprehensive healthcare through the lens of Primary Health Care [33]. Considering both the demand and supply aspects, the insights derived from this study fill the evidence gap essential for policymakers and program administrators to reconsider strategies and devise inventive methods to implement social health insurance in Nepal effectively.

## Material and methods

### Study design

The study utilised a phenomenological approach within an exploratory qualitative research methodology to examine the factors that either facilitate or impede the use of health insurance programs by insured residents of Bhaktapur district in Nepal. To ensure standard reporting, the study followed the consolidated criteria for reporting qualitative studies (COREQ) guidelines[34].

### Study setting

The study was conducted in the Bhaktapur district, which adjoins the capital city Kathmandu in Nepal. It covers an area of 119 square kilometres and is made up of four municipalities. According to the National Census in 2021, the district had a population of 432132, with 218418 being male and 213714 being female. The district had an annual population growth rate of 3.35%, and the average number of family members was 3.98. Regarding the literacy status, 82.26% of individuals can read and write, and 11.11% are illiterate [35]. The average life expectancy in Bhaktapur district was 70.87 years, according to the Human Development Report in 2011 [36].

In fiscal year 2016/17, the National Health Insurance Program was introduced in Bhaktapur district, and as of December 2018, 16,623 households were enrolled in the social health insurance program. Currently, 59 enrollment assistants are employed to enroll the household under the national health insurance program [37]. Since the program’s inception until June 2023, a total of 108,689 households have enrolled in the national health insurance program. In the last fiscal year (2022/23), 17,687 households enrolled in the program, with 10,029 being subsidised families. Additionally, within that year, 38,456 households renewed their national health insurance coverage [25,38]. Bhaktapur has 12 hospitals that offer health insurance services, and it was one of the three districts in the Bagmati Province to implement the program during the second phase of user enrollment [25]. Bhaktapur district was the only district from the Kathmandu Valley that enrolled in health insurance during the second phase of the program expansion. Despite various districts and urban cities being included in this phase, this study’s aim was to focus on assessing urban settings where health facilities are readily available and accessible. As it only consists of urban municipalities, Bhaktapur district was the only district within the Kathmandu Valley fitting this criterion. The findings from this study more precisely represent urban settings. Our objective was to gain insights into the urban context of health insurance implementation. Therefore, this district was purposefully chosen.

### Participants selection

Participants on the demand side were purposefully selected from the community where insurance schemes were implemented. Similarly, service providers were purposively chosen to represent the diverse spectrum of the supply side across various levels of service delivery points. The selection of a diverse set of participants aimed to capture comprehensive perspectives on identifying both enablers and barriers to the insurance scheme.

To ensure maximum variability, purposive sampling [39] was employed for patients, encompassing diverse characteristics such as age, sex, ethnicity, level of education, and other socioeconomic background. A total of eight interviews were conducted with participants from the demand side, while five interviews were conducted with representatives from the supply side, guided by the data saturation theory. In sum, a total of 13 interviews were carried out.

### Data collection

For data collection, a face-to-face interview approach utilising an interview guide was employed [40].To ascertain the validity of the interview guide, consultations were conducted with content experts from the Health Insurance Board, Health Insurance research experts, qualitative research experts, and faculty members from the School of Public Health at Patan Academy of Health Sciences. Prior to the actual interviews, the guidelines underwent a piloting phase, resulting in separate guidelines tailored for insurance service users, non-users, and key informants. The questions embedded in the interview guidelines were designed to discern facilitators and barriers to utilising health insurance services, along with soliciting recommendations for improvement.

Data collection spanned from 01 September 2019 to 15 November 2019. Most interviews with the insured population were conducted in their homes, while key informants were interviewed at their workplaces or preferred locations. Interviews were conducted in Nepali by the authors, SuG (MPH) and SaG (MPH), female MPH students who are proficient in qualitative interviewing in the Nepali language. They were guided by one female supervisor, RAS (MDS, MPH) and two male supervisors, SP (MSc PH) and DRS (MSc, MA), who are experienced in conducting qualitative studies and publications. All three supervisors are academicians. There were no prior relationships between interviewers and participants, and participants were informed about the research objective and consent processes. None of the participants approached face-to-face for the interview and refused to participate in the study. The interview was conducted in a confidential environment without the presence of non-participants during the interview. The interviews ranged in duration from 10 minutes to 40 minutes. Participants provided informed written consent, and their responses were recorded on a password-protected audio-recorded device. Along with the audio record, note was also taken during the interview. To enhance conformability and credibility, member checking was employed, involving probing questions and frequent rephrasing during interviews to ensure information accuracy. Post-interviews, researchers engaged in discussions with participants to validate comprehension of conveyed ideas and get their feedback. The research participants did not undergo repeated interviews. The number of interviews was guided by data saturation theory.

Subsequently, recorded Nepali-language interviews were transcribed and translated into English by researchers. To safeguard anonymity, codes were assigned to interviewees, and confidentiality was maintained by storing interviews in a password-protected folder on a laptop. An independent research team member verified English translations for quality and completeness.

### Data analysis

The data were analysed using thematic network analysis [41]. The verified transcripts in the English language were imported into RQDA packages of EZR software [42], and codes were generated. The analysis of the translated data followed a thematic inductive approach [40] and involved an iterative process of coding, analysis, writing, diagramming, and revision. Common codes were combined, and codes providing similar meanings were merged. Broad themes were generated based on the codes generated. Similar basic themes were combined to create broader organising and global themes that made the excerpts more meaningful. The thematic network analysis was used to group these organising themes into global themes, resulting in a comprehensive exploration of a specific issue. (S1 Table)

**Fig 1:**
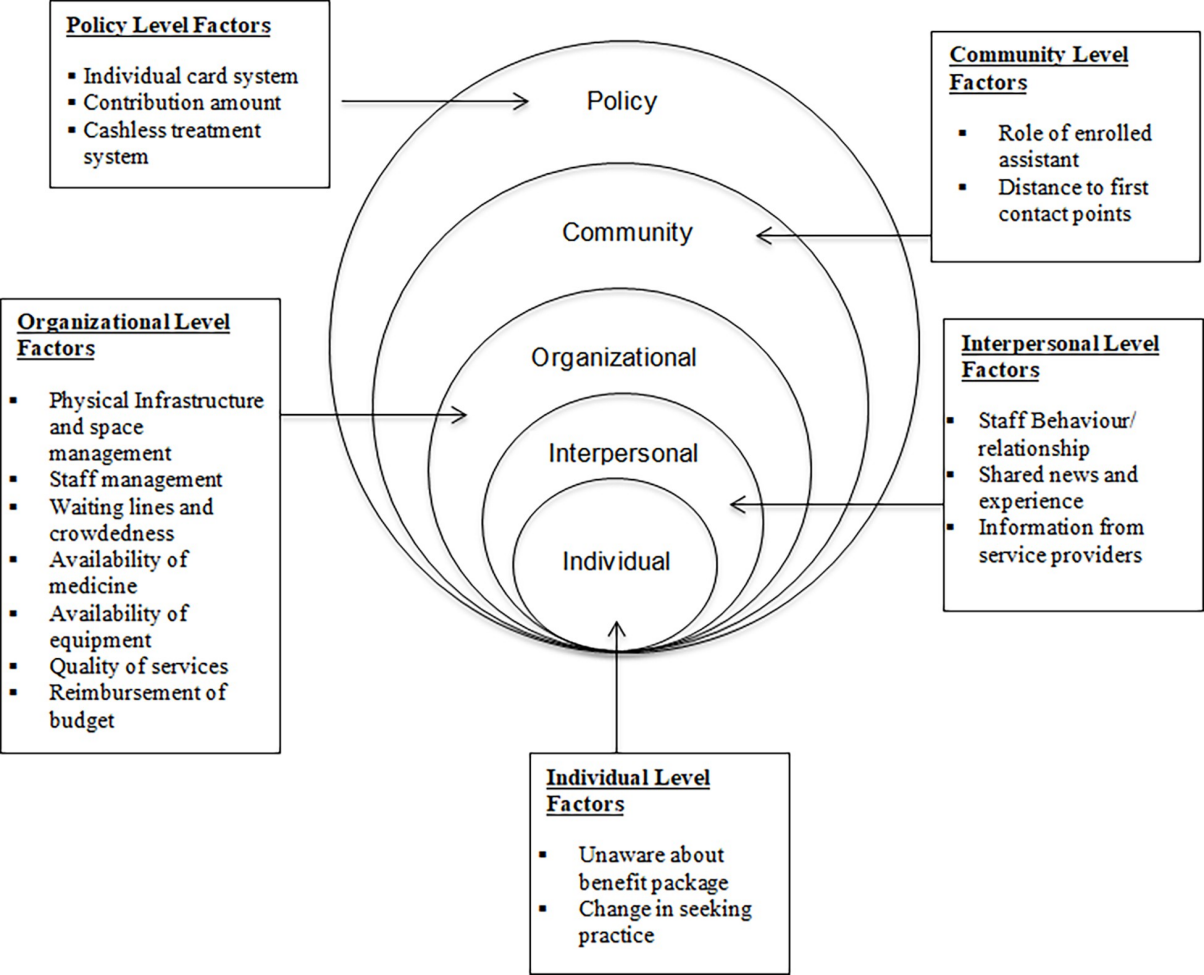
Socio-ecological model on factors affecting utilisation of health insurance service.

Two authors independently coded the translated interview texts to ensure intercoder reliability. The inter-coder reliability was determined to be 77% agreement. The coding process involved two coders, and all the codes from the second coder were found to match the codes from the first coder. The discrepancies in the codes were further discussed jointly among the authors, and findings were presented based on their relevancy. The ecological model was used for the analysis of the factors influencing the utilisation of health insurance services, reflecting its increasing popularity in the field of public health [43]. This model acknowledges the complexity of health-related behaviours, considering individual, interpersonal, community, organisational, and policy-related factors [44,45]. Given the intricate nature of health insurance utilisation, this model allows for an in-depth exploration of how these factors interact. By investigating these factors at various levels, the study provides a comprehensive insight into the drivers and obstacles affecting the utilisation of insurance programs (Fig 1).

The findings and interpretations were presented to selected study participants, who agreed with the results presented, thereby testing the findings. The trustworthiness of the data was ensured through conformability, credibility, dependability, and transferability [40]. Confidentiality and anonymity of the research participants were maintained at all stages of the research process.

### Ethical consideration

The Institutional Review Board under the Nepal Health Research Council at the Patan Academy of Health Sciences granted ethical approval for the research protocol under Reference Number PHP1908091288. The objectives of the study were explained to all the municipalities of Bhaktapur District and the Health Insurance Board, and approval was obtained. Written informed consent was obtained from literate participants, while biometrics (thumbprints) were obtained from illiterate participants as witnesses of literate family members of the participants. The informed consent form was read out aloud prior to the interview, particularly for those illiterate participants. The research adhered to the ethical review guidelines of the Nepal Health Research Council. Participants were fully informed about the research, including its significance, potential benefits and harms, and their right to withdraw from the study at any time without giving any reason or justification. To ensure privacy and confidentiality of the data provided, the researcher kept the collected audio files and translated documents highly confidential. No personal identifiers were used for the participants in the study.

## Results

### Demographic characteristics of study participants

A total of 13 participants, representing both the demand and supply aspects, were engaged in the study to extract the study’s findings. In our pursuit of maximum diversity, we aimed to involve participants across a broad spectrum of gender, age, education, and professional backgrounds (Table 1).

**Table 1:** Demographic characteristics of study participants.

S.N	Sex	Age	Marital Status	Education status	Occupation
1	Female	31–35	Unmarried	Formal education	Service
2	Male	36–40	Married	Formal education	Self employed
3	Female	36–40	Married	Formal education	Service
4	Female	31–35	Unmarried	Formal education	Service
5	Female	56–60	Married	No Formal education	Homemaker
6	Male	51–55	Married	Formal education	Service
7	Female	61–65	Married	No Formal education	Self employed
8	Male	56–60	Married	No Formal education	Self employed
9	Male	41–45	Married	Formal education	Government official at central level
10	Female	31–35	Married	Formal education	Government official at district level
11	Male	31–35	Unmarried	Formal education	Government official at district level
12	Male	51–55	Married	Formal education	Health worker at local level
13	Male	51–55	Married	Formal education	Health worker at local level

*Service includes the employed at any service-oriented jobs (e.g. at a government organization, private or non-government organization).

### Enablers and barriers to utilisation of health insurance services

The aspects that contribute as enablers and obstacles in the utilisation of health insurance services are categorised into five primary themes: Individual-level factors, Interpersonal-level factors, Organisational-level factors, community-level factors and Policy-related factors.

#### 1) Individual level factors

The limited understanding of the benefit package offered by health insurance among those who are insured has been identified as a barrier to accessing health insurance services. Conversely, a change in how individuals seek healthcare has been acknowledged as a catalyst for the utilisation of health insurance services. The study participants have diverse perspectives on their attitudes toward health insurance.

#### Awareness about the benefit package

The service users involved in the study conveyed that their understanding of the services covered by their health insurance benefits was rather limited prior to their actual utilisation of these services. Consequently, they held certain expectations of a broader range of services being encompassed within their benefits package, and these expectations were not consistently met. This discrepancy led to a sense of discontentment with the health insurance services they received. Furthermore, the absence of clear information regarding the requisite procedures for accessing and utilising these services also emerged as a significant impediment. It was acknowledged that the service user’s own lack of familiarity with health insurance proved to be a hindrance in effectively utilising the available health insurance services. One of the service users stated that;

*“…. We don’t know about the beneficiary package. We don’t know which medicines are under insurance and which are not.”* [Service user, 31–35 years, Female]

One of the health insurance service providers also stressed that;

*“They had a misconception that health insurance covered all medications, but in reality, many drugs for specific medical conditions were not included in the coverage.”* [Health worker at local level, 51–55 years, male]

#### Change in health-seeking practice

The service users have indicated a shift in their healthcare-seeking behavior. Prior to enrolling in health insurance services, their preference leaned towards private health facilities. However, they have now transitioned to choosing government health facilities as their primary source of care. This shift is regarded as a positive factor that encourages the utilization of health services among those with insurance coverage. It aligns with the goals of the health insurance program, which aims to ensure efficient and cost-effective healthcare delivery. This change in preference has the potential to enhance the use of health insurance services by providing insured individuals with convenient access to high-quality medical care. One of the service users expressed his shift in healthcare preference, stating,

*“Although we go to private hospitals initially because of the short waiting time, after launching this good program by the government, I am currently going to Bhaktapur Hospital. I have also gone there to get treatment…”* [Service user,36–40 Years, Male]

#### 2) Interpersonal level factors

The conduct of the staff and the details provided by the service providers were hindrances to the utilisation of health insurance services. There were differing opinions about whether the shared news and experiences related to health insurance acted as barriers or facilitators for service utilisation.

#### Staff behaviour/relationship

A predominant grievance voiced by service users pertains to the conduct of staff within health facilities. A substantial number of respondents conveyed instances of disparate treatment encountered during their pursuit of health insurance services. Participants revealed instances of discrimination by health institutions. Staffs discriminate between insured and uninsured patients. The prevailing sentiment was that staff tended to neglect insured members, subjecting them to prolonged waiting periods for treatment. Notably, no grievances were articulated concerning the behavior of physicians. These observed discriminatory practices significantly dissuade individuals from enrolling in insurance schemes or engender reluctance to avail themselves of services offered under such schemes. One of the service users expressed his experiences as:

*“…behavior of the staff is also not good. I feel like they treat us differently than non-insured. It isn’t the same as doctors. All the doctors are good over there. They behave well and treat the patients well. Whenever I go for treatment, they have been able to diagnose and treat me.”* [Service user, 36–40 Years, Male]

Also, one of the service providers noted that:

*“Staffs speak rudely to insured people, which shouldn’t be done. We should respect the insured members.”* [Health worker at local level, 51–55 years, male]

#### Shared news and experience

The dissemination of information within the community by those who have utilised health insurance services plays a role in influencing service utilisation to some extent. Insured individuals who have not used the services mentioned that they preferred other healthcare centres due to dissatisfaction expressed by service users. On the other side, program enrollment has also increased because of the positive experiences shared by insured members with those who are not insured.

A service non-user provided insight into the experiences of those with insurance coverage, remarking:

*“Those brothers who had been insured went to get treatment, and they were told that the medicines weren’t available there.”* [service non user, 55–60 years, male]

However, one of the district enrollment officers (employee of service purchaser) expressed his observations as:

*“Once insured members take the insurance services related to critical illness and operation, then they share their experience of getting treatment from a health facility with other people. Due to this, now the non-insured people are also willing to enroll in the insurance program as they think it is good.”* [Government official at district level, 30–35 years, male]

#### Information sharing by service provider

Inadequate information sharing about health insurance among users plays a hindering role in shaping health insurance service utilisation. The service users expressed their grievances regarding the lack of adequate information concerning the services covered by benefits packages, the medications included in health insurance coverage, and the remaining funds in the benefit ceiling. These gaps in information contribute to their hesitancy in utilising the services. Service providers argue that the enrollment assistant working under the Health Insurance Board should adequately disseminate this essential information. One of the service providers also claimed that:

*“…in reality, those enrolled assistants should be more responsible for informing all about health insurance to the people. It seems to be inadequate to the public.”* [Health worker at local level, 51–55 years, male]

One of the service users also mentioned that:

*“We don’t know about the beneficiary package. We don’t know which medicines are under insurance and which are not.”* [Service user, 31–35 years, Female]

#### 3) Organisational level factors

At the organisational level, factors such as the poor arrangements of physical infrastructure and space, personnel administration, waiting times, congestion, medicine availability, and budget reimbursement posed obstacles to the utilisation of health insurance services. There were varied opinions regarding whether equipment availability and quality of care acted as facilitators or barriers.

#### Physical infrastructure and space management

Healthcare facilities have been striving to manage their available space for service provision. However, in some of these facilities, constraints related to medicine storage space and insufficient physical infrastructures have affected the utilisation of services by insured individuals. Despite these difficulties, efforts have been made to improvise and ensure the availability of examination rooms for service delivery.

One of the service providers expressed his concerns as;

*“There is a problem with space for medicine storage, so we don’t have enough medicine. Due to the lack of a safe dispensary unit over here, we are unable to distribute the medicines.”* [Health worker at local level, 51–55 years, male]

#### Staff management

The increase in the number of patients after the program’s launch has not been met with a proportional increase in staff members. Moreover, the implementation of the health insurance program in hospitals has led to an addition in administrative duties within these healthcare institutions. In addition to the shortage of human resources, the efficient management of existing resources has been identified as a significant challenge in delivering services effectively to patients and insured members. This issue has substantially impacted the poor utilisation of health insurance services. One of the service users shared her observation as;

*“Someday, there were three people, and someday, only one person was available (for the same task) …response to the client was very slow, and then there were long lines.”* [Service user, 61–65 years, Female]

One of the policymakers also provided his insights:

*“They (staff of the hospital) have to claim to get money after providing services. They have to prepare all the documents which were initially not required. Thus, to do this, they also need some extra staff and other additional things. Also, they are required to do online reporting on time otherwise, they won’t receive payment on time. So, they are required to do things fast.”* [Government official at central level, 41–45 years, male]

#### Waiting lines and crowdedness

As the inflow of patients seeking health insurance services at hospitals has grown, there has been a noticeable increase in both crowdedness and long waiting times at hospitals. This phenomenon has played a role in discouraging insured members from utilising health insurance services, given that it leads to delays in patient care. Furthermore, not only do patients grapple with these challenges, but hospitals themselves are also confronted with difficulties in handling the issue, as it significantly amplifies their workload. One of the service non-users expressed her experiences as:

*“Once, I had gone for a check-up, and because of the long line and over crowdedness, I went to another hospital for treatment. Also, people returning from there expressed disappointment as they didn’t get medicine despite waiting for such a long line.”* [Service non-user, 31–35 years, Female]

The provider agreed with the problem of such a scenario:

*“There is a crowd, and they have to wait in a long queue. They might not get the treatment services immediately as before, and more time may be consumed while taking services.”* [Government officials at district level, 31–35 years, male]

#### Availability of medicine

The primary barrier to the utilisation of health insurance services emerged from the limited availability of medications. This factor also contributed significantly to the discontinuation of insurance memberships among individuals. The consumers voiced strong discontentment regarding the absence of prescribed drugs in the facility’s pharmacy. They expressed dissatisfaction with the limited selection of lower-cost medications while more expensive ones required purchase from external sources. Issues related to supply contracts (tender) and doctors specifying particular brand-name drugs led to challenges in consistently accessing medications from the same manufacturer, eroding public confidence in the quality of medicines. This situation necessitated the availability of multiple brands of the same drugs, a demand that not all primary care or first-contact hospitals could fulfill for various reasons. Some healthcare facilities even cited space constraints for medicine storage as a factor contributing to insufficient medication availability, leading to disappointment among those seeking services. For example;

*“They ask to buy expensive medicines under Health Insurance from outside stores, and they only provide low-cost medicines.”* [Service users, 61–65 Years, Male]

On the other hand, the service provider has expressed that the government procedure is lengthy, and not the same companies were selected in procurement for delivering similar brands of medicines that were being distributed to patients in previous visits. However, they have alternative brands, and patients are reluctant to change the medicine brands every time they visit the doctor.

One of the officials in the district described his observations and experience:

*“Another main complaint we have to listen to frequently is the unavailability of medicines. The combined medicine is very limited in health insurance. Those patients with chronic diseases are taking combined medicines in a single tablet, and people are not being assured about using two tablets for those same combination medicines. Also, it being a government procedure, there is a long process of tender and other procurement procedures. The patient taking the same medicine for a long time may not get the same medicine from the same company due to the tender system. If they receive the medicine from a different company, they feel that the medicine doesn’t work. I don’t know whether it is their psychological perception or it is their reality.”* [Government officials at district level, 31–35 years, Female]

#### Quality of services

The quality of healthcare services has played a dual role, acting as an enabler and a barrier in the utilisation of health insurance services. While specific individuals with insurance coverage have conveyed serenity with the care they’ve received, others have expressed disappointment. Some have noted a gradual decline in the quality of care, which they perceived as superior initially. A substantial portion of service consumers indicated that the quality is acceptable, particularly for minor health issues.

One of the service users shared her experience, stating:

*“It (The service) is good for minor illnesses only but not for major illnesses. For the treatment of minor illnesses, I am satisfied because at least we could get a check-up at nearby health facilities due to health insurance.”* [Service user, 56–60 years, Female]

Other young adult service users noted that:

*“In the starting phase, the quality of service was satisfactory, but now it is definitely not satisfactory.”* [Service user, 31–35 years, Female]

#### Availability of equipment

The addition of equipment in healthcare centres providing health insurance services is an ongoing process. In contrast to the initial stages, there has been a gradual increase in the addition of equipment. Nevertheless, a deficiency of equipment persists in those primary care health facilities, resulting in challenges for patients in accessing services. While the majority of respondents express contentment with the accessibility of diagnostic tests, a subset has raised concerns about the inconsistency between test reports from these facilities and those from other hospitals, which has led to dissatisfaction with laboratory services.

The service users provided their mixed views regarding this as:

*“I have found differences in reports of the same diagnostic test over there (service under health insurance) and outside (other hospitals). Thus, I am not satisfied with the diagnostic test over there.”*[Service users, 51–55 years, Male]

*“The lab services have just opened; the equipment and machine (diagnostic test) over there are also very good*.*”* [Service users, 31–35 years, Female]

On the other hand, service providers claimed the effective functioning of the services.

*“The remaining services are functioning effectively, and the laboratory services are also operating smoothly.”* [Health worker at local level, 51–55 years, male]

The staff at the district health insurance board conveyed his perspective in supporting the above statement as:

*“About the lab services, I haven’t heard any complaints because very expensive treatment services, such as MRI, CT-SCAN, etc., are being made available. In this condition, they are satisfied with the lab services and other services as well.”* [Government officials at district level, 31–35 years, Female]

#### Reimbursement of budget

The delay in obtaining reimbursements for claimed expenses from the health insurance board is seen as a hindrance to service delivery, leading to significant outcomes for the utilisation of health insurance services.

The service providers specifically noted that:

*“The money which we claim should be provided on a monthly or fortnightly basis, it needs to be provided as soon as possible.”* [Health worker at local level, 51–55 years, male]

Another service provider also added that;

*“The payment of medicines should be done on time. If the payment is received early, more people will get medical services.*” [Health worker at local level, 51–55 years, male]

#### 4) Community level factors

The presence of the enrolled assistant and the distance of the first contact points in the community have served as facilitators for the insured members to make use of the health insurance services.

#### Role of enrolled assistant

Within each community or ward, a designated enrollment assistant is assigned the responsibility of conducting home visits to enroll new members and renewing the insurance coverage of existing members in the health insurance program. These enrolled assistants play a crucial role in simplifying the registration and renewal process for community members, providing them with guidance and addressing any questions they may have about utilising health insurance services. As a result, these enrolled assistants within the community have significantly facilitated insured members in accessing and utilising health insurance services.

*“Our enrollment assistants in the community effectively communicate with insured members, providing them with satisfactory responses during their interactions.*” [Government officials at district level, 31–35 years, male]

#### Distance to first contact points

Proximity plays a pivotal role in determining service accessibility. The distance to the primary healthcare facility emerged as a key facilitator in the utilisation of health insurance services. Most participants noted that the first-contact healthcare facility providing health insurance services is conveniently situated near their location, making it easily accessible for them to use these services. However, if they require a referral to another healthcare facility, the issue of distance can pose a challenge in accessing treatment.

The service users expressed their views as;

*“First, the hospital is near us so it is easy to reach there to get treatment.”* [Service user, 51–55 years, Male]

#### 5) Policy level factors

The study participants recognised that three elements within policy-level factors–namely, the individual card system for insured members, the contribution amount, and the cashless treatment system–acted as enablers for the utilisation of the health insurance system.

#### Cashless treatment system

The cashless feature of the health insurance program was the predominant factor motivating the utilisation of health insurance services. After enrolling and paying the annual contribution, every insured family member could access and utilise the specified insurance benefits without the need for cash payments. Additionally, the expansion of services covered by the benefit packages proved to be an enticing feature that encouraged insured members to make use of the services. Therefore, this can be regarded as a facilitator for utilisation.

One of the service users articulated his perspective as follows:

*“The most motivating factor for utilising health insurance services is the cost-free service that we get from here. After paying a premium of only NPR (Nepalese Rupees) 2500/3500(≈ $20 USD/ $ 25 USD) per year, we get free services up to NPR 50000/100000 respectively, if need be.”* [Service user, 36–40 Years, Male]

The staff under service purchaser also conveyed her viewpoints as:

*“Now, after increasing the benefit ceiling up to NPR 100,000 (≈$748USD), they (community people) are being more positive towards health insurance. It encourages people to participate in insurance services.”* [Government officials at district level, 31–35 years, Female]

#### Contribution amount

The insured members in the community expressed satisfaction with the annual contribution they make for health insurance. They found the amount to be quite affordable and manageable.

One of the service users delightfully expressed satisfaction like this;

*“Yes, I am very satisfied with that amount. I will continue to pay the premium even if I have to save money for it without consuming food.”* [Service user, 36–40 Years, Female]

#### Individual card system

As each insured member is provided with their own individual card, and treatment expenses are pre-covered, individuals have the autonomy to make decisions about seeking treatment independently. Possessing the insurance membership card grants them access to healthcare facilities. The cashless service and the individual card system facilitate immediate healthcare-seeking without being hindered by waiting for family decisions or financial deliberations.

One of the officials at district level stressed that:

*“Because in this program, every member has their card, so they don’t have to wait for other’s decisions. They have their card with them so they can go to get services even when there is no one at their home (for financial assistance).”* [Government officials at district level, 31–35 years, Female]

One of the service users expressed her experience in support of the above statement as:

*“Since being insured, I didn’t need to wait for my son or daughter-in-law to take treatment services. I visit myself to the hospital by taking my own card. Neither have I needed to wait for them for money nor for their decision to get treatment.”* [Service user, 56–60 years Female]

## Discussion

The study examines diverse factors impacting health insurance service utilisation across individual, interpersonal, organisational, community, and policy levels. At the individual level, participants’ limited awareness of benefit packages led to dissatisfaction and unrealistic expectations. Shifting seeking practices from private to government healthcare facilities encouraged utilisation. Interpersonally, unequal staff treatment affected usage, while shared experiences influenced program enrollment and utilisation. Organisational factors encompassed challenges like limited infrastructure, staffing, crowdedness, and medicine availability, all influencing utilisation. Community-level aspects included assistants aiding registration and proximity to facilities. Policy-wise, cashless systems, contribution satisfaction, and individual cards promoted service utilisation. These factors, from various levels, collectively shape the utilisation of health insurance services.

The satisfaction of the consumer is the key to the sustainability of health insurance services. In the current study, consumers were mostly dissatisfied with the information provided by the health facilities about health insurance services. A review in Indonesia also showed that less utilisation of the services by subsidised poor members was due to a lack of knowledge regarding benefits[46]. Likewise, the impact evaluation of the Medical Insurance Program in Georgia, USA, has also demonstrated low utilisation because of too little information provided on the benefit packages [47]. A mixed-method analysis among rural veterans of Lowa City, US, has also shown consistent findings that lack of information is a barrier to using the benefits of health insurance [48]. Similarly, an evaluation of the Medical Insurance Program in Georgia, USA, revealed reduced utilisation due to inadequate information provision about benefit packages [47]. This highlights the universal importance of overcoming knowledge barriers for effective health insurance implementation.

The study findings unveiled consumer dissatisfaction with healthcare staff behaviour towards those covered by insurance. Many participants reported encountering unequal and disrespectful treatment during their care. A comparable pattern was observed in a study carried out in the Lao People’s Democratic Republic (Lao PDR), indicating that insured members received less favorable treatment and respect compared to uninsured patients from service providers [49]. The inconsistent findings were obtained in Community-Based Health Insurance (CBHI) services of Bangladesh in which, the staff behavior is one of the most satisfying domain identified [50]. As highlighted in the current study, various factors could attribute to the issues, such as insufficient training and awareness among the healthcare staff regarding the rights and expectations of insured individuals in Nepal. Additionally, administrative hurdles in handling paperwork, lack of accountability mechanisms and lack of supervision may contribute to the observed disparity and dissatisfaction.

The current study recognised the distance to the health facility and the affordable contribution amount as enablers for utilisation of health insurance utilisation. The participants were satisfied with the amount of premium they paid. This finding is aligned with the various studies conducted in Nepal [51]. Comparable results were found in both the Ethiopian study [52] and a mixed-method study in Uganda, where affordability and proximity to health facilities were identified as the primary factors influencing household preferences and the use of health insurance services [53]. In the context of Nepal, promoting the utilisation of health insurance services relies on addressing geographical barriers and ensuring financial feasibility, given that primary health care centres serve as the initial points of contact for accessing the benefits of the insurance.

In the current study, a lack of physical infrastructure and staff management are barriers to the utilisation of services. A similar finding was presented in the study, which states lack of infrastructure and shortage of manpower as a great hindrance to the success of the SHI Scheme in Uganda and Indonesia [54,55]. The consistent findings that were derived from the systematic review to assess the barriers and facilitators of Community-Based Health Insurance (CBHI) in LMICs also suggest that at the point of delivery factors such as the accessibility of facilities, facility environment, and health personnel influenced utilisation [56].

In Nepal, inadequate physical infrastructure, including limitations in equipment, space, and overall facilities, may impact service quality and accessibility, while issues in staff management such as shortages of trained personnel, poor coordination, and inadequate training, can impede efficient healthcare delivery [7,28,32,51]. Addressing these barriers requires investments in healthcare infrastructure and improvements in staff management to enhance overall healthcare utilisation effectiveness in Nepal. The current study has also identified delays in reimbursement of the budget as a barrier to the delivery and utilisation of health insurance services. The finding is further supported by the study in other districts (Kailali, Illam and Baglung) implementing social health insurance programs as the reason for dropping out [26]. Evaluation of Rashtriya Swasthya Bima Yojana (National Health Insurance Program of India for Indian poor) had also generated evidence that the delay in reimbursement of the budget by the program to the provider had resulted in hospitals refusing admission of the insured member. A qualitative interview, which was done to assess the perception and experience of providers and beneficiaries of that program in India, also proved that delay in reimbursement and ambiguity in disbursement are the main reasons behind not delivering health services to the insured member in the hospital [57]. In the current study, it could be attributed to the administrative inefficiencies, bureaucratic processes, or financial challenges within the health insurance system of Nepal.

The findings further explore the factors like quality of services as both facilitators and barriers to utilisation. A quantitative study conducted to assess consumer satisfaction in CBHI of Bangladesh showed that most of the insured members were satisfied with the quality of services they received as being as expected [50]. However, findings in Georgia Medical Insurance demonstrate no improvement in service utilisation because of the inadequate quality of services [47]. The study in Lao also showed the poor quality of care as a source of dissatisfaction and also one of the major reasons for leaving the scheme [49]. However, the evidence from Ethiopia demonstrated that the perceived quality of the services was good compared to the past health insurance scheme [52]. The findings from the current study are in line with the quantitative findings conducted in Ghana [58]. The study from Ghana also indicated more than two-thirds of participants perceived receiving a good quality of services, and the remaining indicated not receiving a good quality of services, which implies both facilitator and barrier for utilization [58].

Unavailability of the medicines and crowdedness & long waiting lines are chief complaints made on. A similar study conducted in three districts (Kailali, Illam, and Baglung) of Nepal to assess the performance of the insurance program has also revealed that the unavailability of medicines and laboratory services is the primary reason behind people losing interest in the insurance field [26]. Studies in Ghana also revealed similar findings as the unavailability of essential drugs and long waiting times as reasons for perceiving the poor quality of services received from the health insurance services field [58]. The findings from Lao also revealed that insured members receive low-quality drugs and slower service regardless of the urgency of health care needs [49]. In the present study, the reason behind such barriers may be attributed to challenges in the supply chain, insufficient healthcare facilities, and inadequate staffing in Nepal. These issues not only affect the quality of care but also contribute to dissatisfaction among individuals seeking healthcare services.

The availability of equipment has also served as both a facilitating factor and a barrier to the use of health insurance services. The study in Laos showed that a lack of equipment was a problem for using health insurance services among insured members [49]. Also, a study from Ethiopia showed patients’ dissatisfaction with the available laboratory services under the health insurance scheme [52].

## Limitations and strengths of the study

This study is subject to certain limitations, notably its pre-COVID-19 context, which raises concerns about its relevance in the current landscape. The pandemic has likely caused shifts in risk perception, illness perception, and overall healthcare behaviors, highlighting the need for a reevaluation or update of the findings to accommodate these changes. Therefore, caution should be taken when interpreting and utilising the results of this study. While many factors identified suggest a need for health system strengthening, it’s important to note that factors identified at the personal level may have been altered by the COVID-19 pandemic. Readers should consider the study’s context, as the researchers aimed to ensure diversity in participant selection based on key demographic characteristics but did not explore other potential factors, areas for future studies to delve into capturing the full range of experiences and perspectives across a diverse population.

However, this study marks a pioneering effort in Nepal as it delves into the facilitators and barriers concerning the utilisation of health insurance services in an urban setting, capturing perspectives from both users and providers. The study employed a meticulous approach to participant selection, ensuring a diverse representation across crucial demographic factors. By specifically targeting individuals with a minimum of one year of enrollment, the research gathered a diverse range of experiences and viewpoints, enriching the comprehension of health insurance service utilization. Identifying the factors from individual to policy level factors provides complete and comprehensive information on the utilization of national health insurance services.

### Policy implication

The study’s findings hold significant importance for policy formulation, offering valuable insights for enhancing the utilisation of national health insurance services, especially in urban areas of Nepal. Through an exploration of perceived facilitators and barriers among service users and stakeholders, the study contributes to identifying the key areas for policy interventions at the federal level and local level health systems as well as the improvements in the health insurance program itself. Policymakers at different levels of government can utilise these findings to tailor strategies that address barriers and capitalize on facilitators, thus fostering increased access to healthcare services and better overall health outcomes within the national health insurance framework.

## Conclusion

Both the demand and supply perspectives highlight comparable elements that impact the utilisation of health insurance services. The recognition of various factors, such as cashless treatment systems, distance to the health facilities, individual card systems, contribution amounts, etc., that facilitate or impede the utilisation of these services has implications for the sustainability of national health insurance program. Most of the identified obstacles, such as availability of medicine, staff management, crowdedness and long waiting hours, staff behaviour, and information shared about benefit packages, are concentrated at the point of service delivery, indicating a need for comprehensive quality improvement in care and the reinforcement of the service delivery mechanism. Addressing these recognised barriers is crucial to ensure the effective implementation and realisation of the health insurance program’s aims and its progress towards achieving Universal Health Coverage (UHC). Some identified hindrances stem from inherent issues, particularly the availability of medicine, physical infrastructure, and quality of services, which are linked to the healthcare system of the country rather than the shortcomings of the health insurance program itself. Consequently, enhancing the overall healthcare delivery system holds the potential to enhance the utilisation of health insurance services. This study provides evidence for improving Nepal’s national health insurance implementations, emphasising accessibility, quality, awareness, collaboration, and monitoring. Future studies can focus on post-COVID-19 influences, barriers among marginalised groups, program effectiveness, and exploration of wider perspectives of the program implementation challenges for better policy outcomes.

## Supporting information

S1 Checklist COREQ checklist. (PDF)

S1 Table Thematic Network Analysis Framework based on Socio-ecological model (from codes to global themes). (DOCX)
