# Supplementary material for: Factors influencing the utilisation of National health insurance program in urban areas of Nepal: Insights from qualitative study
Source: PLOS Glob Public Health. 2024 Jul 26;4(7):e0003538. doi: 10.1371/journal.pgph.0003538 (PMC11280150; doi:10.1371/journal.pgph.0003538)
Supplement: S1 Table — (DOCX) [file pgph.0003538.s002.docx]

S1 Table: Thematic Network Analysis Framework based on Socio-ecological model (from codes to global themes)

| **Codes** | **Basic themes** | **Organizing themes** | **Global themes** |
| --- | --- | --- | --- |
| - Insurance - Benefit packages - Unknown about services under benefit packages - Negative attitude to health insurance - Positive attitude to health insurance - No require to wait family member - Self-decide to seek treatment - Own self card - Seeking private hospital - Seeking contact point hospital - Positive experience of treatment under health insurance - Rude behaviour of staff - Behaviour of doctor - Cheap medicine - Different medicine - Lack of expensive medicine - Lack of space for medicine storage - Over crowdedness - Long waiting lines - Equipment - Old buildings - Buildings under construction - Lack of information shared by enrollment assistant - Near health facility - Inadequate staff - Affordable contribution amount - Free contribution amount for old - Cashless treatment system - Free cost of services | 1. Insuree unaware about the benefit packages 2. Positive attitude towards health insurance 3. Negative attitude towards health insurance 4. Self-decide while seeking treatment 5. Tendency to seek hospital under health insurance 6. Inappropriate staff behaviour 7. Staff adequacy and management 8. Good experience shared in a community 9. Bad news and experiences heard 10. Lack of sharing information regarding benefit packages 11. Poor buildings 12. Less space for storing medicine 13. Less space to manage crowdedness and waiting lines 14. Unavailability of expensive medicine 15. Unavailability of same medicine 16. Delay reimbursement of budget 17. Positive role of enrollment assistant 18. Negative role of enrollment assistant 19. Short distance to health facility 20. Individual card system while seeking treatment 21. Appropriate contribution amount 22. Contribution amount versus benefit packages 23. Cashless treatment system | - Unaware about benefit package , change in seeking practice - Staff Behaviour, shared news and experience, lack of information from service providers - Poor physical Infrastructure and space management, Poor staff management, long waiting lines and crowdedness, unavailability of medicine, availability of equipment, quality of services, reimbursement of budget - Role of enrolled assistant, distance to first contact points, - Individual card system, contribution amount, cashless treatment system | 1. Individual Level Factors 2. Interpersonal Level Factors 3. Organizational Level Factors 4. Community Level Factors 5. Policy Level Factors |
